# The XIST lncRNA is a sex-specific reservoir of TLR7 ligands in SLE

**DOI:** 10.1172/jci.insight.169344

**Published:** 2023-09-21

**Authors:** Jonathan D. Crawford, Hong Wang, Daniela Trejo-Zambrano, Raffaello Cimbro, C. Conover Talbot, Mekha A. Thomas, Ashley M. Curran, Alexander A. Girgis, John T. Schroeder, Andrea Fava, Daniel W. Goldman, Michelle Petri, Antony Rosen, Brendan Antiochos, Erika Darrah

**Affiliations:** 1 Division of Rheumatology, Department of Medicine;; 2 The Single Cell and Transcriptomics Core, Institute for Basic Biomedical Sciences; and; 3 Division of Allergy and Clinical Immunology, Department of Medicine, Johns Hopkins University School of Medicine, Baltimore, Maryland, USA.

**Keywords:** Autoimmunity, Immunology, Autoimmune diseases, Innate immunity, Lupus

## Abstract

Systemic lupus erythematosus (SLE) is a systemic autoimmune disease with a dramatic sex bias, affecting 9 times more women than men. Activation of Toll-like receptor 7 (TLR7) by self-RNA is a central pathogenic process leading to aberrant production of type I interferon (IFN) in SLE, but the specific RNA molecules that serve as TLR7 ligands have not been defined. By leveraging gene expression data and the known sequence specificity of TLR7, we identified the female-specific X-inactive specific transcript (XIST) long noncoding RNA as a uniquely rich source of TLR7 ligands in SLE. XIST RNA stimulated IFN-α production by plasmacytoid DCs in a TLR7-dependent manner, and deletion of *XIST* diminished the ability of whole cellular RNA to activate TLR7. XIST levels were elevated in blood leukocytes from women with SLE compared with controls, correlated positively with disease activity and the IFN signature, and were enriched in extracellular vesicles released from dying cells in vitro. Importantly, XIST was not IFN inducible, suggesting that XIST is a driver, rather than a consequence, of IFN in SLE. Overall, our work elucidated a role for XIST RNA as a female sex–specific danger signal underlying the sex bias in SLE.

## Graphical abstract

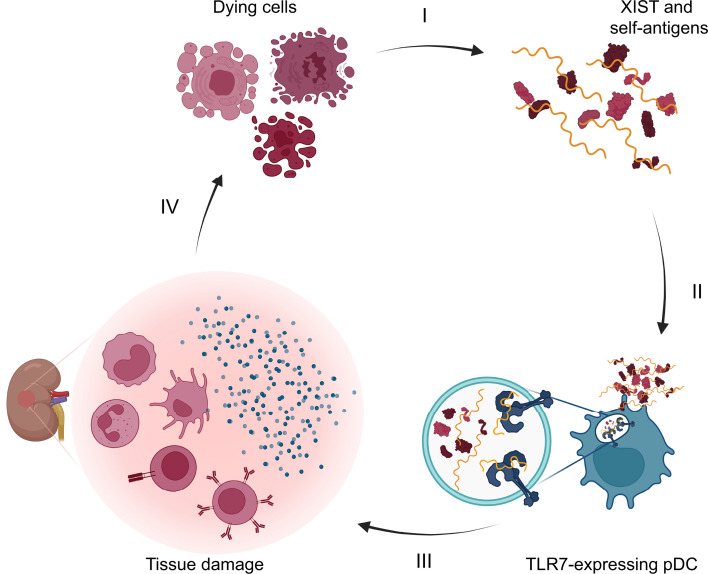

## Introduction

Female sex is associated with enhanced function of both innate and adaptive immune pathways, including interferon (IFN) production and Toll-like receptor (TLR) activation (1–4). As a result, female individuals have improved viral defense and vaccine responses (2, 5–7), but this enhanced immune function is accompanied by a greatly increased risk of autoimmunity compared with male individuals (8–10). A key molecule at the intersection between the enhanced protective and autoreactive immune responses in women is the pattern recognition receptor TLR7. TLR7 is a sensor for single-stranded RNA (ssRNA) encoded by a gene on the X chromosome (11–13). Mounting evidence demonstrates that *TLR7* escapes X chromosome inactivation (XCI), leading to enhanced expression of *TLR7* in women compared with men (14–16). Overexpression of or gain of function in *TLR7* is causal to the development of systemic lupus erythematosus (SLE) in humans and mouse models (16–19). SLE has emerged as one of the most female sex–biased autoimmune diseases, affecting 9 times more women than men (20–22), and is characterized by immune-mediated damage to multiple organ systems (23) and the prominent upregulation of genes induced by type I IFN, known as the IFN signature (24). Aberrant recognition of self-RNA by TLR7 is central to this phenomenon (25), but it is unknown whether female sex–specific self-RNAs ligate TLR7 and thus contribute to the sex bias of SLE. Identification of female sex–specific TLR7 ligands would suggest that women harbor elevated levels of endogenous adjuvants that synergize with the enhanced expression of *TLR7* to amplify TLR7-dependent immune responses.

Given the strong female bias in SLE and central role of self-RNA in stimulating TLR7-dependent immune responses in this disease, we hypothesized that female-specific self-RNAs harboring TLR7-stimulatory motifs may be critical partners in SLE pathogenesis. The sequence specificity of TLR7 has recently been elucidated, and it is clear that TLR7 can recognize both pathogen-derived and self-RNA (i.e., endogenous) sequences (26, 27). Recent structural work has revealed that most nucleotide sequences that bind the ssRNA recognition site of TLR7 are 3- to 5-mers containing UUX, where X is preferentially C or U but can be any nucleotide (28, 29). Ligand-receptor binding experiments have revealed a variety of UU-containing sequences to bind TLR7 with affinities between 100 (CCUUCC) and 680 nM (GGUUGG), with many UU-containing sequences still unexplored (28). In addition, Hornung et al. identified a 20-mer region of a self-RNA transcript, termed RNA9.2s, that stimulated IFN-α production by plasmacytoid dendritic cells (pDCs) in a *TLR7*-dependent manner (12). Mutational analysis highlighted the importance of the 3′ 9 nucleotides of RNA9.2s (i.e., 5′-GUCCUUCAA-3′) for TLR7 activation. Based on this evidence, we sought to identify sex-biased self-RNA transcripts that contained putative TLR7 ligands and examine their TLR7-stimulatory capacity and relevance to SLE pathogenesis.

Our study identified the X-inactive–specific transcript (XIST), a long noncoding RNA (lncRNA) known for its canonical role in XCI, as a female-specific source of TLR7 ligands. We show that XIST RNA is a uniquely UU-rich, highly abundant, and female sex–specific lncRNA that also harbors the GUCCUUCAA motif, rendering it a potent inducer of TLR7-dependent responses, even in the context of whole cellular RNA. This pro-inflammatory role for XIST RNA in disease pathogenesis in SLE was supported by our finding that XIST RNA levels are elevated in SLE and associated with both the IFN signature and clinical disease metrics. Our finding that *XIST* expression is not induced by IFN strengthens the conclusions that elevated XIST levels in SLE are a cause rather than a consequence of the IFN signature. Together, these studies reveal that XIST acts as a female-specific danger signal that, in combination with its receptor TLR7, may contribute to the sex bias in SLE (30–32).

## Results

### XIST RNA is rich in putative TLR7 ligands.

**Figure 1:**
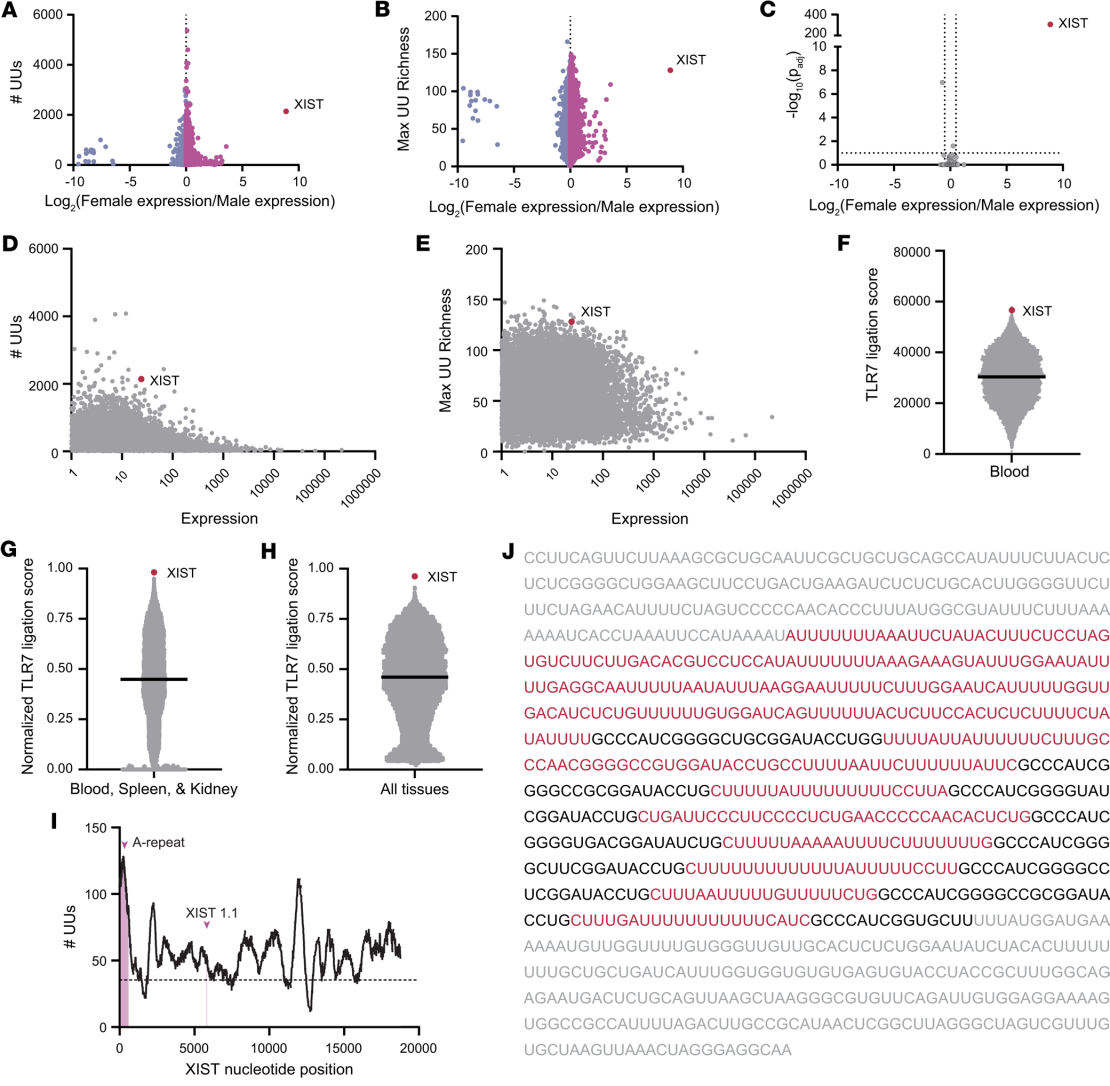
XIST is a sex-biased source of putative TLR7 ligands. (**A**) Dot plot showing the number of UU dinucleotides and degree of sex bias in expression of all 15,003 transcripts captured in publicly available RNA-sequencing samples from blood (33). (**B**) Dot plot showing the maximum local (500-nucleotide) UU richness and degree of sex bias of all transcripts in **A**. (**C**) Volcano plot showing the sex bias of transcripts from **A** that also contain the known TLR7 ligand 5′-GUCCUUCAA-3′ (33). (**D**) Dot plot showing the number of UU dinucleotides and expression level of all transcripts in **A**. (**E**) Dot plot showing the maximum local (500-nucleotide) UU richness and expression of all transcripts in **A**. (**F**) Dot plot showing each transcript’s rank sum score calculated based on the rank of each transcript’s number of UUs, maximum UU richness, degree of female sex bias, and expression level. (**G**) Dot plot showing the average normalized rank sums for each transcript in tissues of particular interest in SLE (blood, spleen, and kidney). (**H**) Dot plot showing the average normalized rank sums for each transcript in all tissues. (**I**) A line chart indicating the local UU richness surrounding each point in the XIST sequence. The number of UUs in the 500-base section starting with each nucleotide is shown. The A-repeat region and XIST1.1, the region containing the 5′-GUCCUUCAA-3′ motif, are denoted. The dotted line indicates the average UU richness of all transcripts in the human transcriptome. (**J**) The first 1,000 nucleotides of the XIST sequence, which contains the A-repeat region, are shown. The A-repeats are shown as black text, with the surrounding UU-rich regions highlighted as red text. Nucleotides outside the A-repeat region are shown as gray text. (**A**–**C**) Differential expression testing was performed using empirical Bayes estimation with multiple comparisons corrections inherent.

We hypothesized that important sources of TLR7 ligands in SLE would be self-RNA transcripts with a female sex bias, that are rich in known TLR7 stimulatory motifs, and that are highly expressed. To identify sex-biased transcripts rich in TLR7 ligands, we first performed differential expression analysis between female and male donors on 175 peripheral blood samples from the Genotype-Tissue Expression (GTEx) database (33) (Supplemental Table 1; supplemental material available online with this article; https://doi.org/10.1172/jci.insight.169344DS1). We quantified the total number of UU dinucleotides in each transcript and plotted UU count against the sex bias in each transcript’s expression, defined as the log_2_ fold-change in female over male expression (Figure 1A). We found that XIST, a 19 kb lncRNA responsible for mediating XCI, demonstrated the most pronounced female expression bias and UU dinucleotide enrichment, as it contained 2,140 UU dinucleotides and was expressed at 472 times higher levels in the female than male sex (Figure 1A). Since transcript length is a critical factor for the total number of UU dinucleotides a transcript may have, we also considered that short UU-dense transcripts or regions of transcripts could also be key TLR7 activators. Therefore, we also calculated the highest UU density in any 500-base segment of each transcript, termed the “maximum UU richness,” and plotted this metric versus the sex bias for each transcript (Figure 1B). Again, the XIST RNA emerged as containing the highest UU richness of any sex-biased transcript in the blood, with 128 UU dinucleotides in a 500-base region.

In addition to UU dinucleotide content, extended nucleotide sequences around the UU motif may allow for enhanced TLR7 binding. Given the known TLR7-stimulatory potential of the 5′-GUCCUUCAA-3′ motif within the RNA9.2s transcript Hornung et al. identified (12), we also searched for sex-biased transcripts containing this known TLR7-stimulatory motif. Of the 293 transcripts containing this motif, the XIST RNA was found to be the only sex-biased source of this motif (Figure 1C).

We next considered that transcript abundance is likely critical for the importance of any transcript as a source of TLR7 ligands, since more abundant transcripts would be expected to contribute a higher number of potential TLR7 ligands within the context of whole cellular RNA. To search for abundant UU-rich transcripts, we plotted transcripts per million (TPM) expression values in female samples of all transcripts expressed in blood versus the number of putative TLR7 ligands in each transcript (Figure 1D). In this analysis, we found that only nuclear enriched abundant transcript 1 had a higher expression and more UU dinucleotides than XIST. By plotting expression against the maximum UU richness for each transcript, we again found only 1 transcript, sorting nexin 2, with higher expression and a more UU-rich region than XIST (Figure 1E). Last, when looking at the 293 transcripts containing the 5′-GUCCUUCAA-3′ motif, we found XIST to be more highly expressed than 84% of transcripts containing this motif in blood (Supplemental Table 1). To evaluate the overall weight of evidence for a given transcript being a TLR7 ligand, we ranked each transcript found in the blood based on each of 4 criteria from low to high: female expression bias, total UU count, maximum UU richness, and expression. We then calculated the sum of each transcript’s rank for the 4 criteria and termed this sum the “TLR7 ligation score,” with a higher score representing a higher potential for being a TLR7 ligand. XIST had the fourth highest rank sum TLR7 ligation score, putting it in the top 0.03% of transcripts (Figure 1F). The sex bias, UU counts, max UU richness, presence of GUCCUUCAA, differential expression analysis results, and rank sum analysis results for blood are provided in Supplemental Table 1.

We repeated this rank sum analysis for all tissues available in the GTEx database, including tissues of interest in SLE (i.e., spleen and kidney), and found XIST to also be remarkable in its ubiquity. XIST had the second highest TLR7 ligation score by rank sum in spleen, had the third highest in kidney, and was a top-10 candidate in every other tissue analyzed except muscle (Supplemental Table 2). Overall, averaging the normalized TLR7 ligation scores of all tissues analyzed revealed XIST as the top candidate by TLR7 ligation score when looking at tissues of interest in SLE (blood, spleen, and kidney; Figure 1G) or all tissues (Figure 1H). The expression levels, results of differential expression testing between male and female samples, total UU count, maximum UU richness, and presence of the 5′-GUCCUUCAA-3′ motif for every transcript in spleen and kidney are included in Supplemental Table 3, A and B, and the rank scores for each transcript in each tissue are provided in Supplemental Table 2.

To identify specific regions within the XIST RNA molecule with the highest potential to act as TLR7 ligands, we examined the XIST sequence more closely. We found that most of the XIST sequence is more UU-rich than the average transcript in the human transcriptome and that the maximally UU-rich region was located in an area known as the A-repeat region (Figure 1I). The A-repeat region, which is essential for the canonical function of XIST in XCI, supports the DNA binding and multimerization of the XIST molecule (34) and is composed of short A-repeat sequences connected by long U-rich linkers (Figure 1J). While the A-repeat region is near the 5′ end of XIST between nucleotides 181 and 1,007, the known TLR7 stimulator 5′-GUCCUUCAA-3′ motif, which we termed XIST1.1, resides in the middle of exon 1 at nucleotides 5,824–5,843, indicating that multiple regions of the XIST RNA may have the capacity to ligate TLR7. Together, our unbiased search for sex-biased TLR7 ligands revealed the XIST lncRNA as the strongest candidate source of female-specific TLR7 ligands in the human genome.

### XIST RNA contains TLR7 ligands that stimulate human pDCs and HEK-hTLR7 reporter cells.

**Figure 2:**
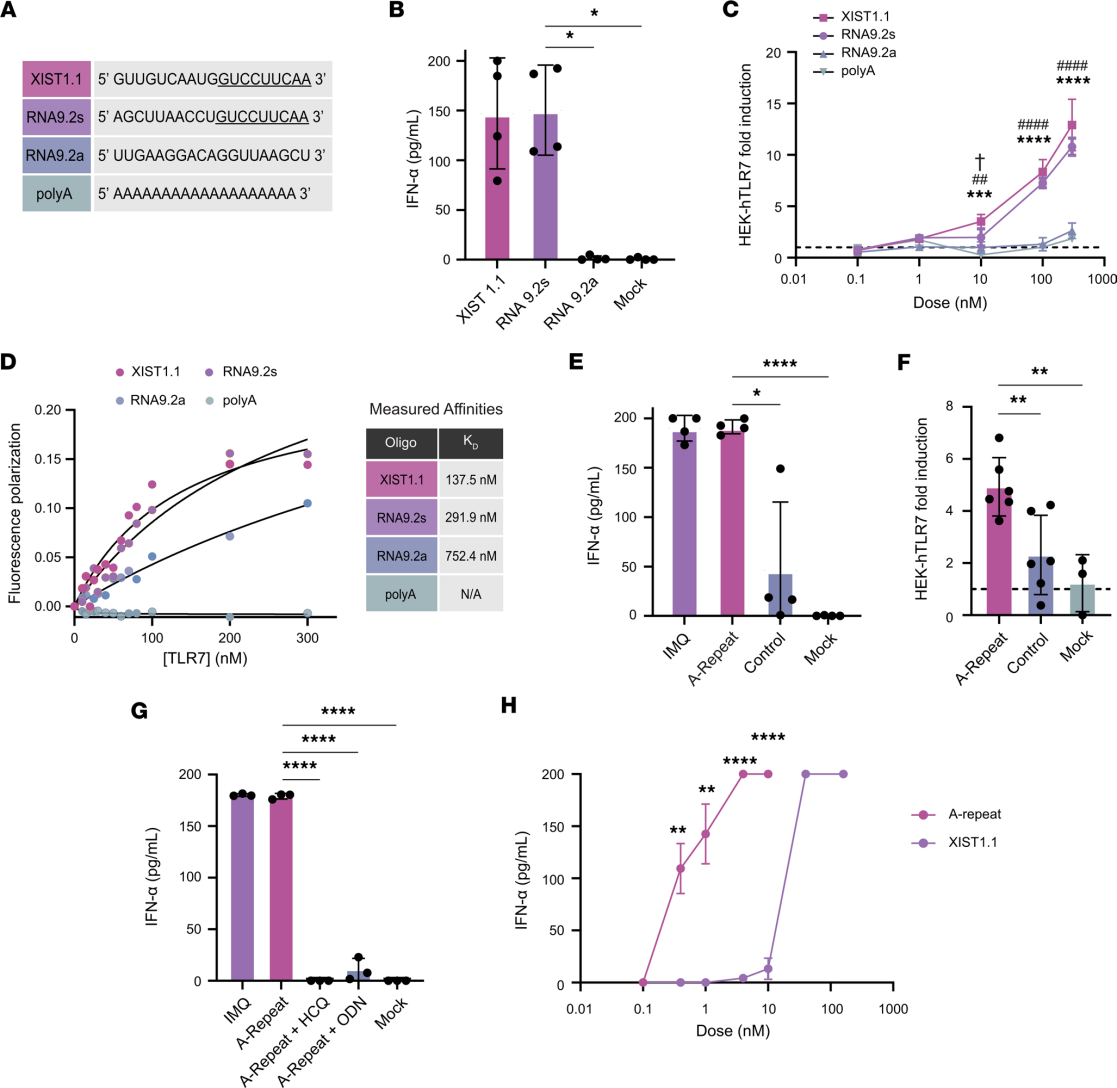
TLR7 ligands in XIST activate human pDCs and HEK-hTLR7 cells. (**A**) Sequences of the 4 RNA oligonucleotides used to stimulate pDCs and HEK-hTLR7 cells. (**B**) ELISA measuring IFN-α production by pDCs from 4 different healthy donors after transfection with 20-mer oligonucleotides. (**C**) Colorimetric assay showing the production of SEAP by HEK-hTLR7 cells in response to treatment with 20-mer oligonucleotides in 3 technical replicates. All treatments were compared with XIST1.1 by multiple comparisons at each dose. † Indicates XIST1.1 versus RNA9.2s, ^#^ indicates XIST1.1 versus RNA9.2a, and * indicates XIST1.1 versus polyA. One symbol indicates *P* < 0.05, 2 symbols indicate *P* < 0.01, 3 symbols indicate *P* < 0.001, and 4 symbols indicate *P* < 0.0001. (**D**) Fluorescence polarization values of XIST1.1, RNA9.2s, RNA9.2a, and polyA RNA when incubated with increasing doses of human recombinant TLR7. (**E**) ELISA measuring IFN-α production by pDCs after transfection with A-repeat or control RNA from 4 different healthy donors. IMQ was used as a positive control. (**F**) Colorimetric assay showing the production of SEAP by HEK-hTLR7 cells after transfection with A-repeat or control RNA in 6 technical replicates. (**G**) ELISA measuring IFN-α production by pDCs after transfection with A-repeat with or without TLR7 inhibitors HCQ or ODN from 3 different healthy donors. (**H**) ELISA measuring IFN-α production by pDCs after transfection with varying concentrations of A-repeat RNA or XIST1.1 in 3 technical replicates. A-repeat was compared to XIST1.1 by *t* test at each dose. (**B**–**H**) Error bars indicate 1 standard deviation. * Indicates *P* < 0.05, ** indicates *P* < 0.01, *** indicates *P* < 0.001, and **** indicates *P* < 0.0001. (**B** and **E**–**G**) All treatments were compared with XIST1.1 or A-repeat by 1-way ANOVA with multiple comparisons correction.

To investigate whether the fragment of XIST containing the 5′-GUCCUUCAA-3′ motif can act as a TLR7 ligand, we measured IFN-α production by pDCs in response to transfection with 3 RNA 20-mers: a) XIST 1.1, the fragment of XIST RNA containing the 5′-GUCCUUCAA-3′ motif at the 3′ end; b) RNA9.2s, the sequence that was originally identified as a TLR7 motif by Hornung et al. (12); or c) RNA9.2a, the nonstimulatory antisense oligo of RNA9.2s (Figure 2A). We found that the XIST1.1 fragment stimulated robust IFN-α production by pDCs that was equivalent to the response to the positive control, RNA9.2s (Figure 2B). RNA9.2a and mock transfection did not induce IFN-α production, as expected.

To verify that this was a TLR7-dependent effect, we used HEK-hTLR7 reporter cells, which stably express human *TLR7* and release secreted embryonic alkaline phosphatase (SEAP) into the culture medium in a NF-κB–dependent manner downstream of TLR7 signaling. By treating HEK-hTLR7 reporter cells and the untransfected parental line (HEK-null) with increasing doses of imiquimod (IMQ), a known TLR7 ligand, we validated that the reporter cells were specifically stimulated via TLR7 ligation in a dose-dependent manner (Supplemental Figure 1A). We then treated the HEK-hTLR7 cells with increasing concentrations of XIST1.1, RNA9.2s, RNA9.2a, or polyA RNA as an additional negative control. Marked dose-dependent activation of TLR7 by XIST1.1 and RNA9.2s was observed (Figure 2C), with significantly more TLR7 activation observed in response to 10, 100, and 300 μg/mL XIST1.1 RNA compared with equal concentrations of RNA9.2a or polyA RNA, indicating that the XIST1.1 fragment is a potent TLR7 ligand.

To verify that the TLR7 activation and IFN-α production observed were due to receptor-ligand binding interactions between the 20-mer RNAs and the TLR7 receptor, we measured binding affinities for the RNA/TLR7 complexes using fluorescence anisotropy. XIST1.1 and RNA9.2s were observed to bind strongly to TLR7, with measured affinities (and 95% confidence intervals) of 137.5 nM (74.82–270.2 nM) and 291.9 nM (152.6–724 nM), respectively (Figure 2D). These affinities are similar to those measured for 6-mer RNA oligos identified as ligands for TLR7 via crystallography (28). On the other hand, RNA9.2a RNA, which did not stimulate TLR7 in pDCs or HEK-hTLR7 reporter cells, had an affinity for TLR7 of 752.4 nM (341.7–6565 nM, significantly lower than that of XIST1.1), and polyA RNA had no demonstrable affinity for TLR7. These findings were verified via an electrophoretic mobility shift assay (EMSA) that showed that coincubation of TLR7 protein with XIST1.1 resulted in the most complex formation with TLR7, followed by 9.2s, and 9.2a, with polyA RNA unable to bind to TLR7 (Supplemental Figure 1, B and C).

Given that we identified the UU-rich A-repeat as a distinct region of XIST, which contains over 100 putative TLR7 ligands in a short 500-base region, we hypothesized that this region would be a potent inducer of TLR7 activation in addition to XIST 1.1. We thus investigated the capacity of the XIST A-repeat region to stimulate TLR7-dependent responses using HEK-hTLR7 reporter cells and primary human pDCs. To do this, we used in vitro transcription to produce a 815 bp transcript containing the A-repeat region of XIST and synthesized a comparable-length fragment of peptidylarginine deiminase 4 (PAD4) as an irrelevant control RNA (Supplemental Figure 1D). We then transfected pDCs with XIST A-repeat RNA or PAD4 control RNA, performed a mock transfection, or treated the pDCs with IMQ as a positive control. We found that pDCs produced an average of 4.14-fold more IFN-α in response to XIST A-repeat RNA than to control RNA (*P* = 0.02; Figure 2E). By transfecting HEK-hTLR7 reporter cells with the A-repeat and control RNA, we again observed that the XIST A-repeat RNA was significantly more stimulatory than equal-length control RNA (*P* = 0.007; Figure 2F).

To further verify the TLR7 dependence of the IFN-α response to XIST A-repeat RNA, we transfected human pDCs with A-repeat RNA in the presence of hydroxychloroquine (HCQ), an endosomal TLR inhibitor, or ODN20958 (ODN), a TLR7-specific inhibitor. We found that both inhibitors blocked IFN-α production by pDCs in response to the A-repeat region of XIST, confirming that XIST RNA is activating pDCs in a TLR7-specific manner (Figure 2G). Finally, to investigate the relative abilities of the XIST1.1 and A-repeat regions to act as TLR7 ligands, we performed a dose titration with equimolar concentrations of each RNA fragment between 100 pM and 160 nM. We found that the A-repeat RNA, with over 100 UU dinucleotide motifs, was a more potent TLR7 ligand than the smaller XIST1.1 fragment, capable of inducing half-maximal IFN-α production by pDCs at a concentration that was over 25-fold lower than XIST1.1 (0.4 vs. 10–40 nM; Figure 2H). These results verify that XIST RNA sequences are able to stimulate TLR7-dependent production of IFN and highlight that the UU-richness of XIST, rather than the 5′-GUCCUUCAA-3′ motif, is most important for its function as a TLR7 ligand.

### XIST depletion substantially reduces the amount of TLR7 ligand in female cellular RNA.

**Figure 3:**
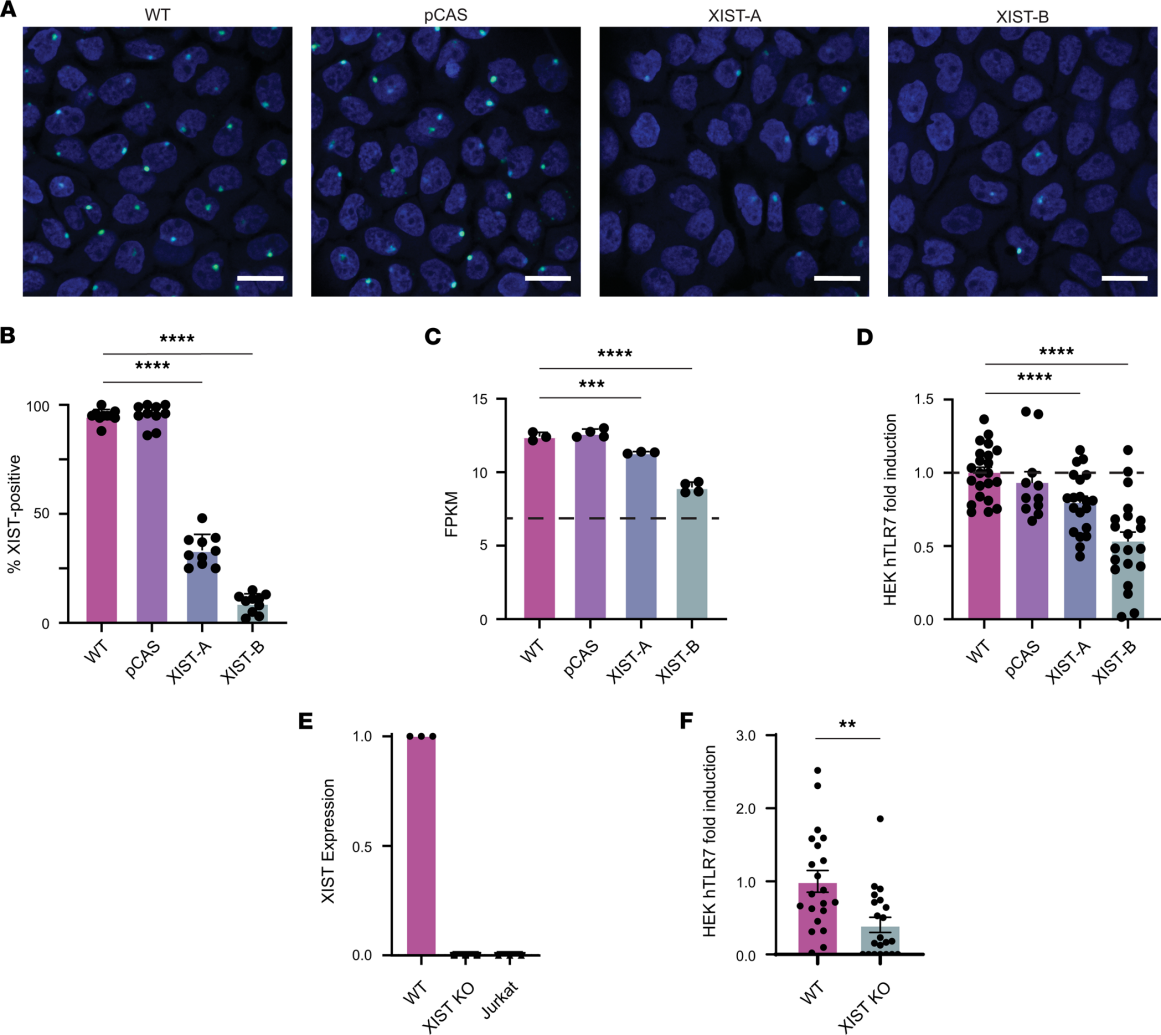
*XIST* knockdown diminishes TLR7-activating potential of whole cellular RNA. (**A**) A431 cells were targeted with CRISPR/Cas9 technology to generate *XIST*-depleted XIST-A and XIST-B cell populations. Representative RNAScope images showing XIST expression (green) and nuclei (blue) in WT, pCAS, XIST-A, and XIST-B cultures. Scale bar indicates 20 μm. (**B**) Percentage of XIST-positive cells was determined by masked quantification of 10 randomly chosen RNAScope image fields per cell population. (**C**) Bar graph showing *XIST* expression in terms of fragments per kilobase of exon per million mapped fragments (FPKM) in each cell population as measured by RNA sequencing. The dotted line at *y* = 7 shows the lower limit of detection in our RNA-sequencing experiment. (**D**) Colorimetric assay measuring SEAP secretion by HEK-hTLR7 cells transfected with fragmented cellular RNA from WT, pCAS, XIST-A, or XIST-B cells. Results shown are pooled from 3 independent experiments. (**E**) Bar graph showing XIST expression in WT, XIST-KO, and Jurkat cells as measured by qPCR. XIST expression calculated using the ΔΔCt method using GAPDH as an internal control and the WT cell line as the reference. (**F**) Colorimetric assay measuring SEAP secretion by HEK-hTLR7 cells transfected with fragmented cellular RNA from either WT or XIST-KO A431 cells. (**B**–**F**) All conditions compared with WT by multiple comparisons within 1-way ANOVA with multiple-comparison correction. ** indicates *P* < 0.01, *** indicates *P* < 0.001, and **** indicates *P* < 0.0001. (**F**) Conditions compared by unpaired *t* test. (**B**–**F**) Error bars represent 1 SD.

We next sought to determine the contribution of cell-derived XIST RNA to TLR7 activation in the context of whole cellular RNA. To do this, we used CRISPR/Cas9-based gene editing to generate XIST-depleted cell populations using the A431 cell line (a female-derived, epithelial cell line that expresses XIST) and to visualize *XIST* expression before and after depletion by RNAScope. Since XIST performs XCI by binding to the inactive X, XIST is typically found in a single punctate Barr body in cells with 2 X chromosomes. In wild-type A431s (WT), we found that nearly all cells contained an accumulation of XIST at a single Barr body. Importantly, treatment with scrambled guide RNA (pCAS) did not affect *XIST* expression. After 1 round of treatment with CRISPR guide RNAs targeting *XIST*, RNAScope revealed a mixed population in which 67% of cells were XIST negative (Figure 3, A and B). We termed this population of cells XIST-depleted population A, or XIST-A. In order to further reduce the amount of *XIST* expression in the population, an additional round of CRISPR/Cas9-based editing was utilized, resulting in another mixed population in which 91% of cells were XIST negative, which we termed XIST-depleted population B, or XIST-B.

To investigate whether potentially important transcriptional changes occurred in response to XIST depletion, we performed RNA sequencing on all 4 A431 cell populations. This analysis verified a reduction in the amount of *XIST* expression by the XIST-A and XIST-B cell populations (Figure 3C). We also looked broadly at transcriptional changes between the WT and XIST-B cell populations, analyzing the change in expression of all 11,675 genes measured with high confidence, where high confidence was defined as FPKM ≥ 7.0 (Supplemental Figure 2A). We considered genes that experienced a change in expression > 4-fold and a Benjamini-Hochberg–adjusted *P* value < 0.05 to be significantly differentially expressed between these 2 cell populations. We found only 63 such genes (0.05%), including *XIST*, that met this criteria. As expected, given the role of epigenetic modification in maintaining XCI for most genes postembryogenesis (35), XIST depletion did not result in preferential reactivation of X-linked genes (Supplemental Figure 2B).

To determine whether other potentially important sources of TLR7 ligands were lost in the XIST-B cell population, we plotted the total number of UUs for every gene downregulated more than 4-fold in the XIST-B cell population versus its expression in WT A431 cells (Supplemental Figure 2C). As expected, XIST was the most UU-rich transcript downregulated in the XIST-B cell population, followed distantly by semaphorin 5A, which had fewer than half as many UUs as XIST, suggesting minimal nonspecific perturbation of UU-rich RNA levels. We also searched the transcripts of all 63 genes whose expression was reduced for the 5′-GUCCUUCAA-3′ motif and found that only 2 other genes (*SCNN1G* and *SCNN1B*) contained this motif. Importantly, however, both genes were expressed at lower levels in the WT cell line (FPKM = 7.96 and FPKM = 7.08, respectively) than *XIST* (FPKM = 12.44). Therefore, we concluded that XIST RNA was the most important source of TLR7 ligands that differed between the WT and XIST-B cell populations.

We then investigated the stimulatory potential of the XIST RNA in HEK hTLR7 reporter cells, when considered in the context of the total cellular RNA isolated from each of the 4 A431 cell populations (i.e., WT, pCAS, XIST-A, and XIST-B). To mimic the natural phenomenon of TLR7 being activated by RNA degradation products (28), we performed Mg^2+^ fragmentation of the A431 RNA prior to transfection. Fragmented RNA was between 25 and 50 bases in length (Supplemental Figure 2D). Using a titration of WT RNA from 1–400 ng, we found that transfection with 100 ng of cellular RNA optimally induced TLR7 activation in HEK-hTLR7 reporter cells (Supplemental Figure 2E). We therefore selected 100 ng as the optimal dose at which to evaluate the effect of changes in XIST RNA level on TLR7 stimulation. Strikingly, we found a dose-dependent effect of *XIST* expression on HEK-hTLR7 cell activation. Compared with WT RNA, the ability to activate TLR7 was reduced 21% with XIST-A RNA and 47% using XIST-B RNA (Figure 3D). The stimulatory capacity of pCAS control RNA was not significantly different from WT RNA in this assay.

The dose-dependent reduction in TLR7-stimulatory capacity of whole cellular RNA correlating with a decrease in the proportion of XIST-positive cells in the population suggested the XIST RNA as a major source of TLR7 ligands in female cells. To precisely quantify the contribution of XIST RNA to TLR7 stimulation, we performed single-cell selection on the XIST-B cell populations to isolate *XIST*-knockout (XIST-KO) cells. Complete loss of *XIST* expression in the *XIST*-KO cells was verified by quantitative PCR (qPCR) (Figure 3E). Transfection of HEK hTLR7 reporter cells with fragmented RNA from WT and *XIST*-KO cells revealed that XIST RNA accounted for 67% of the TLR7-stimulatory capacity of female cell RNA, with significantly less TLR7 signaling observed in HEK hTLR7 reporter cells treated with *XIST*-KO cellular RNA (*P* = 0.0012; Figure 3F). Together, these experiments support our hypothesis that the XIST lncRNA is a potent source of sex-specific TLR7 ligands, with this single RNA comprising a substantial fraction of the stimulatory capacity of female cellular RNA when considered in the context of the entire transcriptome of female cells.

### XIST RNA levels are elevated in patients with SLE and correlate with clinical disease activity.

Given the striking ability of the XIST RNA to stimulate TLR7-dependent IFN-α production in vitro, we investigated whether there was evidence for an immunostimulatory role for the XIST RNA in patients with SLE. Our hypothesis that the sex-specific expression of this endogenous TLR7 ligand predisposes women to systemic autoimmunity suggests that XIST RNA levels may stratify women based on their risk for SLE development. To measure XIST RNA levels in human peripheral blood mononuclear cells (PBMCs) from women with SLE and sex-matched controls, we first optimized an RNA flow assay to detect XIST RNA at the single-cell level by flow cytometry. We validated this approach by performing parallel RNA flow cytometry and qPCR on cell lines with known *XIST* expression levels: Jurkat, a male T cell leukemia line with low levels of XIST, and HEK293T, a female embryonic kidney cell line with high levels of XIST due to the presence of 2 inactive and 1 active X chromosomes per cell (36). We verified the expected expression of XIST in these 2 cell lines and found strong agreement between qPCR (Supplemental Figure 3A) and RNA flow cytometry (Supplemental Figure 3B), with HEK293T cells expressing orders of magnitude more XIST RNA than Jurkat cells in both assays. Next, we validated the ability of RNA flow to quantify *XIST* expression by biological sex in PBMCs from 12 healthy female and 10 healthy male donors and found that women expressed significantly higher levels of XIST RNA than men (*P* < 0.0001), as expected. Importantly, the housekeeping gene *RPL13A*, which resides on chromosome 19, did not exhibit this female sex bias (Supplemental Figure 3C).

**Figure 4:**
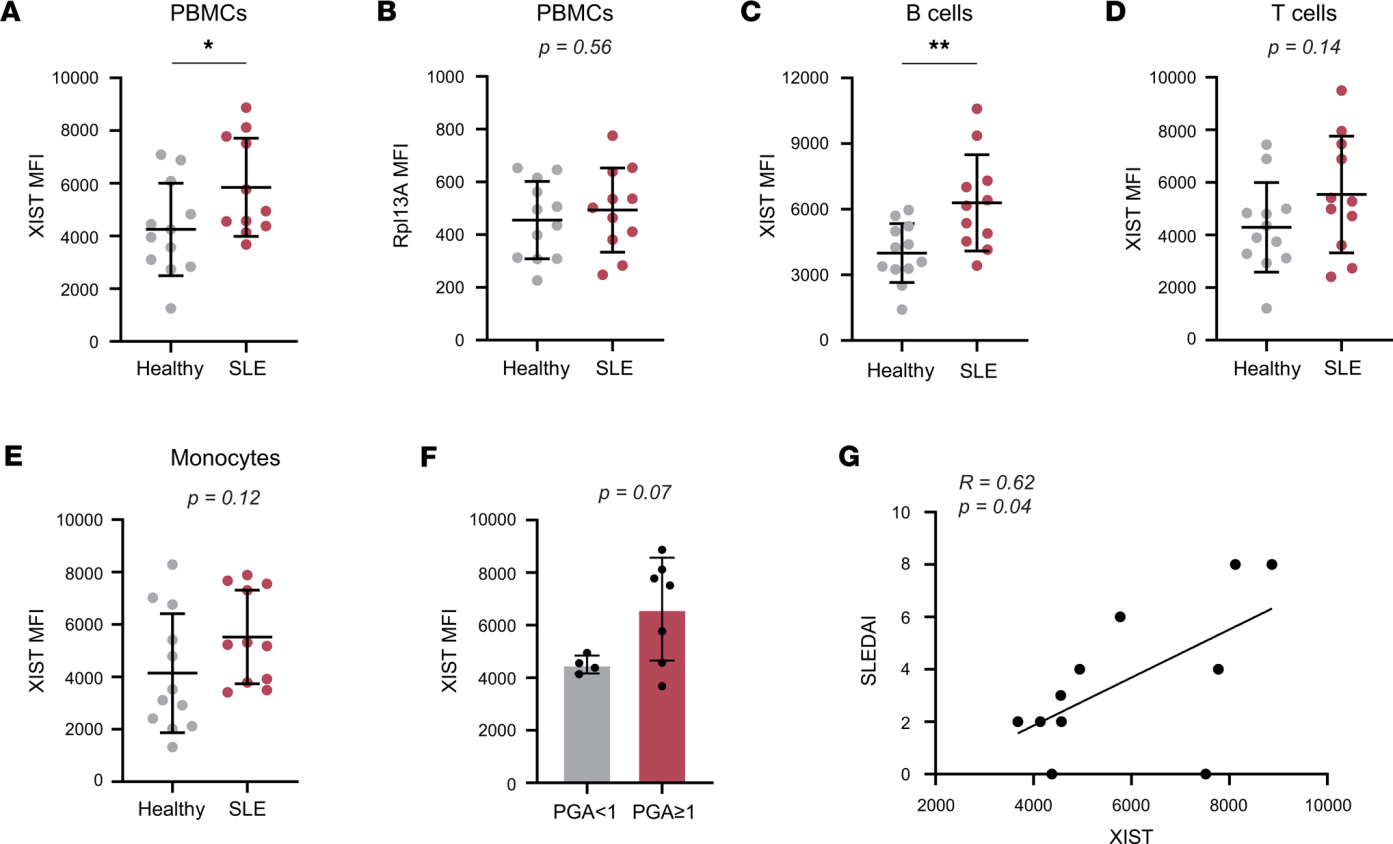
*XIST* expression in PBMCs correlates with SLE status and disease activity. RNA flow cytometry was used to measure *XIST* and *Rpl13A* expression levels in PBMCs from women with SLE (n = 11) versus healthy women (*n* = 12). (**A** and **B**) Dot plots showing XIST (**A**) and RPL13A (**B**) MFI in SLE and control PBMCs. Healthy controls are also shown in Supplemental Figure 3C. (**C**–**E**) XIST MFI in B cells (**C**), T cells (**D**), and monocytes (**E**) from patients with SLE and controls. (**F**) Bar graph showing XIST MFI in SLE patients with a PGA ≥ 1 (*n* = 7) and a PGA < 1 (*n* = 4). (**G**) Scatterplot of XIST MFI versus SLEDAI. Pearson’s correlation coefficient and *P* value for linear regression shown. (**A**–**E**) * indicates *P* < 0.05, and ** indicates *P* < 0.01. Error bars indicate 1 standard deviation. (**A**–**F**) Expression of XIST or RPL13A compared between patients with SLE and healthy donors (**A**–**E**) or disease groups (**F**) by *t* test. (**G**) Correlation tested by simple linear regression.

We then used RNA flow cytometry to compare the expression of *XIST* in PBMCs from 11 women with SLE versus 12 healthy women (Figure 4A). Demographic and clinical data are included in Supplemental Table 4, and our gating strategy is shown in Supplemental Figure 4. Using this approach, we found significantly higher levels of XIST RNA in cells from women with SLE compared with sex-matched healthy controls: the MFI ± SEM of XIST in female SLE PBMCs was 5,848 ± 561.6 compared with 4,252 ± 505.9 in female healthy controls (*P* = 0.04). On the other hand, *RPL13A* expression did not differ between patients with SLE and sex-matched controls (Figure 4B). The difference in *XIST* expression was most exaggerated in B cells (*P* = 0.006; Figure 4C), but the trend was also reflected in T cells and monocytes (*P* = 0.14 and *P* = 0.12, respectively; Figure 4, D and E). Importantly, the elevated *XIST* expression in SLE PBMCs was not driven by differences in cellular composition. The percentage of B cells, the highest expressors of *XIST* in patients with SLE, was similar in PBMCs from patients and controls (7.8% vs. 10.7%, *P* = 0.16), as was the percentage of monocytes (15.23% vs. 10.8%, *P* = 0.34), and the percentage of T cells was significantly lower in patient PBMCs compared with healthy controls (32.7% vs. 59.8%, *P* < 0.0001; Supplemental Figure 3D).

The elevated levels of XIST RNA in women with SLE over healthy control women suggest that XIST RNA may play an active role in the disease process. We therefore examined the relationship between *XIST* expression and disease activity, as measured by the physician’s global assessment (PGA) and the systemic lupus erythematosus disease activity index (SLEDAI). We found that patients with active disease (PGA ≥ 1) had an average of 46.9% more XIST RNA in their PBMCs than patients with PGA < 1 (*P* = 0.07; Figure 4F). In accordance with this result, we also observed that *XIST* expression in PBMCs was significantly correlated with SLEDAI, with each point increase in the SLEDAI being associated with a 1,089.7-unit increase in XIST MFI (*r* = 0.62; *P* = 0.04; Figure 4G). Overall, these data indicated that *XIST* expression is elevated in SLE patient PBMCs compared with healthy controls and is increased in patients with higher disease activity.

### XIST RNA is a cause and not a consequence of the IFN signature.

**Figure 5:**
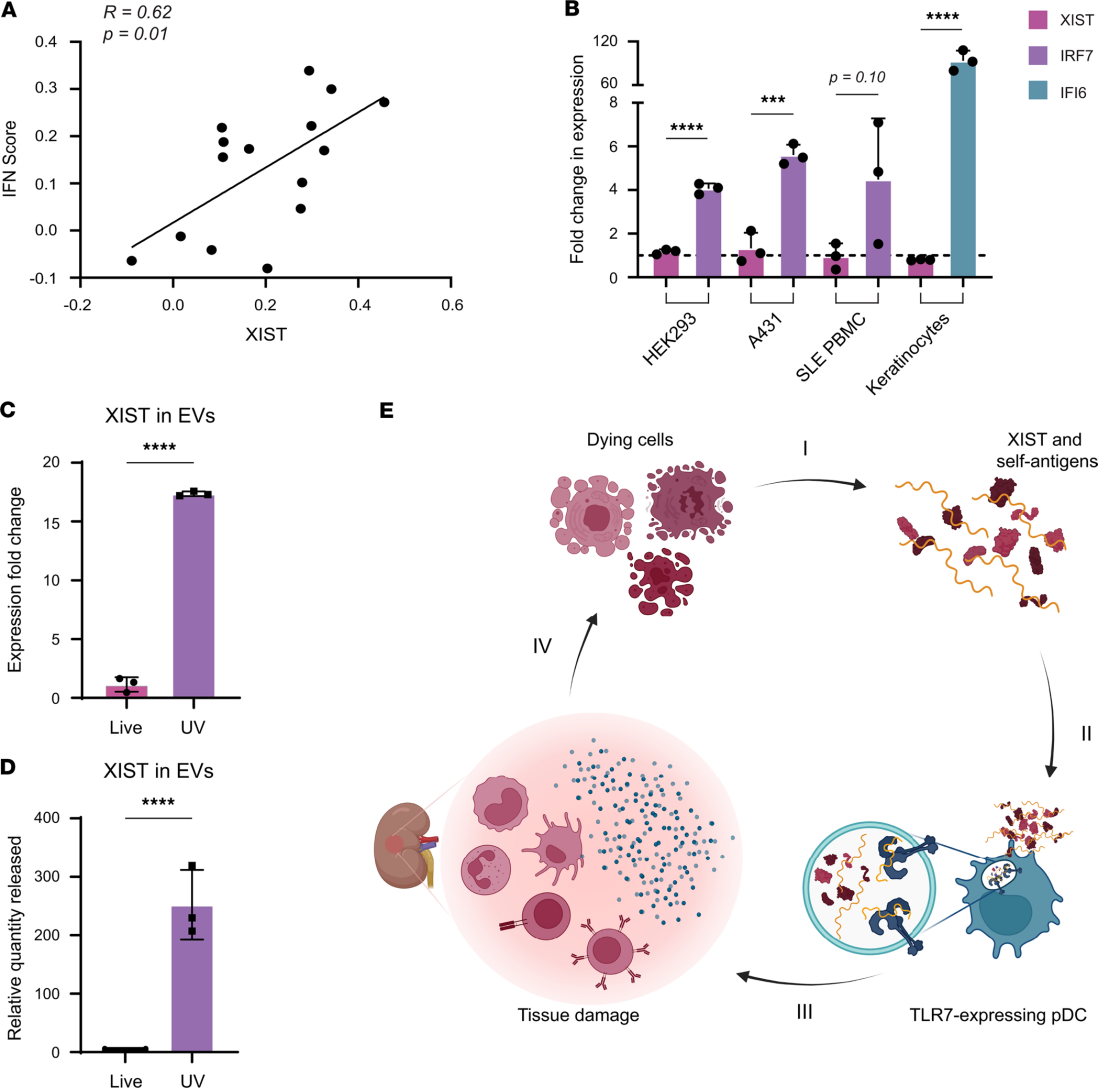
XIST activates IFN production in a cell-extrinsic, TLR7-dependent mechanism. (**A**) Scatterplot showing average *XIST* expression in immune cells from 15 patients with lupus nephritis from the AMP data set versus IFN score (37). (**B**) Bar graphs showing *XIST* and *IRF7* expression in response to IFN-α treatment as measured by qPCR in HEK293 cells, A431 cells, SLE patient PBMCs, and primary human keratinocytes. Upregulation of *IRF7* versus *XIST* or *IFI6* versus *XIST* compared by *t* test. *** indicates *P* < 0.001, and **** indicates *P* < 0.0001. (**C** and **D**) Bar graphs showing *XIST* expression fold-change calculated using the ΔΔCt method relative to *GAPDH* (**C**) or the relative quantity released (**D**) of *XIST* in EVs, calculated as the fold-change in *XIST* multiplied by the fold-change in the amount of RNA isolated, as measured by qPCR. Release of *XIST* RNA compared by *t* test. **** indicates *P* < 0.0001. (**E**) Our model for the role of XIST in SLE pathogenesis. (I) Dying cells release XIST and self-antigens. (II) XIST RNA activates TLR7 in pDCs. (III) Activated pDCs make large amounts of IFN, inducing the IFN signature and an inflammatory environment in the target tissue. (IV) Inflammatory environment and lymphocyte infiltration leads to increased cell death, inducing more XIST release to perpetuate the cycle. (**B**–**D**) Error bars represent 1 SD.

Coupled with our in vitro findings, the observations that XIST RNA levels are higher in women with SLE and correlate with disease activity suggests a model whereby XIST RNA released from dying cells is engulfed by TLR7-expressing immune cells and stimulates IFN production in SLE. To interrogate this hypothesis, we first examined whether XIST RNA levels were associated with evidence of IFN production in the SLE target tissue using single-cell RNA-sequencing data from kidney-infiltrating immune cells in patients with lupus nephritis available through the Accelerating Medicines Partnership (AMP) (37). Production of type I IFN is the output of signaling downstream of TLR7 ligation, and the IFN signature is a hallmark feature of SLE indicating in vivo exposure to IFN. For each cell in the AMP data set, we calculated an IFN response module score as previously described (37). For each woman with SLE in the data set, we then calculated the average IFN response score and compared it with the average *XIST* expression level. This analysis showed that XIST RNA levels strongly correlated with the IFN response module score (*r* = 0.62, *P* = 0.013; Figure 5A), providing clear evidence for a positive relationship between *XIST* expression and IFN signaling. To evaluate the rigor and specificity of this method, we also performed this analysis for all other genes in the data set and found that only 2.69% of genes (604/22,448) were positively correlated with the IFN signature, including many known IFN-responsive genes. The full list of genes positively and negatively correlated with the IFN signature is provided in Supplemental Table 5, A and B.

To further examine the causal relationship between XIST RNA levels and IFN, we next evaluated whether *XIST* expression was induced by IFN. To examine this, we treated HEK293 cells, A431 cells, primary female keratinocytes, and PBMCs from SLE patients with IFN-α and measured *XIST* expression pre- and posttreatment. In all cell types tested, we found that IFN-α increased the expression of known IFN-stimulated genes (either interferon regulatory factor 7, *IRF7*; or interferon-gamma inducible protein 16, *IFI6*), by 4.1- to 80-fold, but did not significantly affect *XIST* expression. *XIST* expression was relatively static after IFN-α treatment, with a 1.17-fold induction in HEK293 cells, a 1.33-fold induction in A431 cells, a 0.96-fold induction in SLE PBMCs, and a 0.80-fold induction in primary human keratinocytes (Figure 5B). These data indicate that the relationship between XIST RNA levels and IFN-α production is likely unidirectional, with XIST acting as an inducer of IFN-α rather than as an IFN-stimulated gene.

Since extracellular vesicles (EVs) released during apoptosis are a major source of extracellular RNA implicated in SLE pathogenesis, we hypothesized that trafficking of XIST to EVs during apoptosis is important for its role as a danger signal (38, 39). We used qPCR to measure XIST levels in EVs from live WT A431 cells and UV-irradiated WT A431 cells undergoing apoptosis. We found that apoptosis induced the trafficking of XIST to EVs, with EVs from apoptotic A431 cells containing 17.36-fold more XIST than live A431 EVs, using GAPDH as an internal control (Figure 5C). These changes were due to an increase in the amount of XIST found in the EVs as opposed to preferential degradation of the internal control RNA (GAPDH), as the relative quantity of XIST in EVs released by apoptotic A431 cells was increased by 252.3-fold over those from live EVs (Figure 5D). The increase in XIST RNA levels in EVs during apoptosis indicated that XIST is trafficked into apoptotic vesicles during cell death, which have a well-established role in SLE and are known to contain many SLE autoantigens. Taken together, these data support our model that XIST RNA acts as an endogenous female-specific danger signal that is packaged into EVs upon cell death and stimulates TLR7-dependent secretion of IFN by pDCs, thus contributing to the development of SLE and increased disease activity, as depicted in Figure 5E.

## Discussion

Activation of TLR7 signaling by self-RNA is central to the current understanding of SLE pathogenesis, with prior work elucidating its importance in mouse models and human studies (17, 18, 23, 25, 40). Yet, the identity of specific RNA molecules responsible for TLR7 activation in this setting remains obscure. Our investigation reveals the XIST lncRNA as a rich reservoir of endogenous TLR7 ligands that is unique to females and overexpressed in women with SLE compared with sex-matched healthy controls. In our unbiased transcriptome-wide survey, XIST was unique in its exceptional female bias, high level of expression, and density of putative TLR7 ligands including the UU-rich A-repeat region and the well-characterized TLR7-activating 9-mer in exon 1 (12, 28, 29, 41). Our in vitro studies verified the sizeable contribution of XIST-derived TLR7 ligands to TLR7 signaling and IFN-α production by pDCs, findings that were mirrored in kidney-infiltrating and peripheral blood leukocytes from women with SLE, in which XIST RNA levels were correlated with the IFN signature and disease activity, respectively. Notably, the sex of our pDC donors was unavailable and may be an important factor in TLR7 activation by XIST given that female pDCs may have higher levels of TLR7 than male pDCs (14). Furthermore, while our experiments focused on pDCs because they are major producers of IFN in SLE, B cells are also known to rely on TLR7 activation for activation and antibody production, and the effect of XIST RNA on B cells also warrants further investigation. Our findings suggest that the XIST RNA acts as an endogenous female-specific danger signal that equips female cells with an enhanced capacity to stimulate TLR7-dependent immune responses. This quality may contribute to the female immunologic advantage in response to both viral infections and vaccines, while lowering the threshold for the development of autoimmune diseases, including SLE.

This hypothesis is supported by the observation that men with Klinefelter syndrome (XXY) and women with triple X syndrome, who have high levels of *XIST* expression due to their having additional X chromosomes, demonstrate an increase in both antiviral responses and incidence of SLE (20, 42, 43). While many genes reside on the X chromosome that could increase SLE risk, *XIST* is one of the few genes that is not dose normalized between XXY men and XY men, since it is not subject to X inactivation (44). As observed in our study, enhanced *XIST* expression in the peripheral blood cells from women with SLE was also reported in a small study by Zhang et al., who performed RNA sequencing of T cells from 5 patients and 12 healthy controls (41). However, our analysis of the GTEx data revealed that *XIST* is ubiquitously expressed, indicating that XIST RNA could be supplied by many cell types, including immune cells, epithelial cells, or stromal cells.

Our study was unique in its analysis of XIST levels in relation to clinical variables and revealed a positive correlation between XIST levels in PBMCs and SLE disease severity, suggesting an amplifying role for XIST in SLE pathogenesis. Moreover, our analysis of gene expression in lupus nephritis biopsies revealed a positive correlation between *XIST* expression and the IFN signature, yet we did not find *XIST* itself to be an IFN-stimulated gene. These findings implicate a unidirectional relationship in which XIST RNA is a driver of enhanced IFN production in females, rather than a product of increased IFN signaling. In this way, XIST RNA may contribute to the observed higher responsiveness of women to TLR7 ligands compared with men (42). The elevated levels of XIST RNA in men with Klinefelter syndrome, women with triple X syndrome, and women with SLE, as well as the association of XIST RNA levels with SLE disease activity, suggests a disease-amplifying role for XIST outside of its canonical role in XCI, with a dose-dependent relationship between XIST levels and SLE risk. Future work in animal models of SLE or in those overexpressing *XIST* will be important to confirm the unidirectional relationship between *XIST* expression and disease development.

The canonical role of XIST RNA is to initiate XCI, and dysregulated XCI has been implicated in SLE pathogenesis. Recent work has shown that XIST is necessary to limit expression of *TLR7* and restrain atypical B cells, which are expanded in SLE (15). In addition, biallelic expression of *TLR7* via partial escape from XCI in the B cells of women and XXY men has also been shown, leading to enhanced *TLR7* expression in individuals with 2 X chromosomes (14, 15, 45). Given these observations, our work suggests that both the ligand (*XIST*) and its receptor (*TLR7*) are overexpressed in SLE and synergize to drive IFN production.

In addition to the interferogenic role of XIST RNA in stimulating TLR7, XIST may also participate in SLE pathogenesis via binding to protein autoantigens. Given its considerable size and function in the Polycomb repressive complex, XIST binds numerous nuclear proteins, some of which may be autoantigens (46). Indeed, an unbiased ChIRP-MS screen (47) revealed that the SLE and Sjögren’s syndrome autoantigen SSB/La, along with the SLE autoantigen hnRNP A2/B1 (48), interacts with XIST. This finding suggests that XIST-autoantigen complexes may be released by dying cells and endocytosed into TLR7-containing endocytic compartments by antigen-presenting cells in the SLE microenvironment. These XIST-autoantigen complexes would then have the capacity to induce the production of IFN, as well as provide both signal 1 (i.e., peptide antigen for CD4^+^ T helper cell recognition) and signal 2 (i.e., costimulatory signals upregulated in response to TLR7 ligation), leading to activation of T helper cells and autoreactive B cell differentiation into autoantibody-producing plasma cells. XIST-autoantigen recognition by autoantibodies could amplify this process, leading to the formation of RNA-containing immune complexes in the setting of cell death, which are known to induce IFN production and antigen presentation by pDCs, B cells, and conventional DCs (49–51). The recent observation that XIST is found in atypical subcellular localization patterns in lymphocytes suggests that human immune cells may be a source of XIST-autoantigen complexes (52). Determining whether XIST is present in immune complexes and identifying additional XIST-binding autoantigens in SLE are important areas for future study.

The identification of XIST as an endogenous sex-specific source of immunostimulatory TLR7 ligands unites 2 longstanding observations in SLE — the presence of the IFN signature and the disproportionate incidence in individuals with 2 or more X chromosomes (9, 20–22, 24, 37, 42, 43, 53). The identification of XIST as a danger signal is distinct from its previously defined role in XCI (14, 15, 17, 52). Our data suggest that not only is the TLR7 receptor overexpressed in women and Klinefelter men compared with XY men, but so is the XIST RNA ligand, dramatically increasing the potential for activation of the TLR7 signaling pathway in these groups. This finding has broad implications for understanding the female immunological advantage and the sex bias in autoimmunity.

## Methods

### Identification of important sources of TLR7 ligands in the human transcriptome.

RNA-sequencing expression data were downloaded from the GTEx database, version 8 (33). Briefly, RNA sequencing was performed using a non–strand-specific protocol with polyA selection of mRNA, and gene expression of 20,110 protein-coding genes and 11,790 lncRNAs was quantified, as described before (54).

To count TLR7 ligands in each gene transcript, we used R version 3.6.1 to search the human transcriptome using the biomartr, stringr, and genomes packages (55–58). After downloading the transcriptome from RefSeq (41), a data frame containing each unique gene in the transcriptome and its sequence was created. For genes with multiple sequence variants listed under the exact same gene name, only the longest variant was included. All genes were searched using R for the known TLR7 ligand 5′-GUCCUUCAA-3′. To calculate organ-specific expression levels, we only considered transcripts with matches in RefSeq and excluded genes with fewer than 25 reads in 10% or more of samples. TPM and log_2_(fold-change) values were then computed directly from counts data. Differential expression between males and females was calculated in each of 22 matched tissues using empirical Bayes estimation implemented through EdgeR (59). Sex-specific tissue samples (uterus, vagina, breast, ovary, cervix/uteri, fallopian tube, prostate, and testis) were excluded. False discovery error correction was implemented using the Benjamini-Yekutieli procedure with α = 0.05 in the R package mutoss (60).

To calculate rank sums, each gene was ranked in ascending order by each of the following criteria: a) total UU count, with the transcript with the lowest UU count being ranked 1 and that with the highest UU count being ranked 15,003; b) maximum UU richness; c) strongest male expression bias (ranked 1) to that with the strongest female expression bias (ranked 15,003) based on the fold-change in expression between sexes; and d) TPM. TLR7 ligation score was calculated as the sum of the 4 ranks for each transcript from low to high, with a higher score representing higher TLR7 ligation capacity. To compare XIST expression across multiple tissues, TLR7 ligation scores for each tissue were normalized by dividing them by the highest TLR7 ligation score in that tissue, and then the normalized TLR7 ligation score in each tissue was averaged.

### Generation of XIST1.1, A-repeat, and control RNA.

The following custom oligonucleotides were synthesized (IDT): XIST1.1 (5′-GUUGUCAAUGGUCCUUCAA-3′), RNA9.2s (5′-AGCUUAACCUGUCCUUCAA-3′), RNA9.2a (5′-UUGAAGGACAGGUUAAGCU-3′), and polyA (5′-AAAAAAAAAAAAAAAAAAA-3′). To construct the plasmid containing the 5′ A-repeat region of human *XIST*, a 747 bp fragment (nucleotides 105–851) of human *XIST* was amplified by Platinum SuperFi Green PCR Master Mix (Invitrogen, catalog 12369010) from HEK293 cell cDNA. To prepare HEK293 cDNA, total RNA was extracted from HEK293 cells (ATCC) by TRIzol Reagent (Invitrogen, catalog 15596018), and cDNA was synthesized using SuperScript VILO Master Mix (Invitrogen, catalog 11755050). The following primer pairs were used in the PCR reaction: forward 5′-TCTAGAACATTTTCTAGTCCCCCAACACCC-3′ and reverse 5′-CACACACCACCAAATGATCAGCAGC-3′. The *XIST* PCR fragment was subsequently cloned into the pCR-XL-2-TOPO vector containing a T7 promoter using the TOPO XL-2 Complete PCR Cloning Kit, with One Shot OmniMAX 2 T1 Chemically Competent *E*. *coli* Cells (Invitrogen, catalog K8050-10). A fragment of PAD4, cloned into pEF-DEST51 as previously described (61), was also subcloned into a T7 promoter–containing vector to produce a transcript of similar length as a control. Both plasmids were sequenced by Sanger sequencing by the Johns Hopkins Genetic Resources Core Facility to verify that the T7 promoter was upstream of the gene fragments and that no mutations were introduced by PCR.

XIST A-repeat and PAD4 control RNA were transcribed in vitro using RiboMAX Large Scale RNA Production System – T7 Kit (Promega, catalog P1300). Briefly, template DNA for the reaction was prepared as follows: the A-repeat–containing TOPO plasmid was linearized by digestion with PmeI (New England Biolabs, catalog R0560S), and the plasmid containing human PAD4 was linearized by digestion with Eco53kI (New England Biolabs, catalog R0116S), prior to ethanol precipitation, drying, and resuspension in RNase-free water. In vitro transcription was then performed according to the manufacturer’s instructions, incubating at 37°C for 4 hours. After transcription, the DNA template was degraded using DNase I (Thermo Fisher Scientific, catalog EN0521) at 1 U/μg of template DNA and incubated at 37°C for 15 minutes. Extraction of RNA and chromatographic removal of unincorporated nucleotides were performed according to the manufacturer’s protocol. RNA length (815 bp for the A-repeat RNA and 884 bp for the PAD4 control RNA transcript) was visualized on a 1% agarose gel (100 ng of RNA in Gel Loading Dye, Purple; New England Biolabs, catalog B7024S), and 1 kb Plus DNA Ladder (Thermo Fisher Scientific, catalog 10787018) was used for reference.

### pDC enrichment.

Healthy control PBMCs were isolated from deidentified leukopaks, obtained as medical excess from the Anne Arundel Medical Center, by Percoll density-gradient centrifugation. Prior to pDC isolation, monocytes were depleted from the PBMC fraction using CD14 MicroBeads (Miltenyi Biotec, catalog 130-050-201). pDCs were then isolated from 1 billion monocyte-depleted PBMCs by negative selection using the EasySep Human Plasmacytoid DC Enrichment Kit (Stemcell Technologies, catalog 19062) according to the manufacturer’s instructions, resulting in approximately 5 million pDC-enriched cells per isolation. The sex of the leukopak donors was not available; 3 to 4 donors were used for each experiment; and each experiment was run in triplicate. Donors who did not make detectable IFN-α in response to any condition were excluded from the analysis.

### pDC stimulation assay.

For treatment with A-repeat and PAD4 RNA, 3 × 10^4^ pDCs were plated in each well of a 96-well plate in 100 μL RPMI (Thermo Fisher Scientific, catalog 11875093) and rested 1 hour before transfection. Each well treated was transfected with 0.2 μg RNA using Lipofectamine MessengerMAX reagent (Thermo Fisher Scientific, catalog LMRNA003) according to the manufacturer’s instructions in the presence of 10 U SUPERase-In RNase Inhibitor (Invitrogen, catalog AM2696). After transfection, pDCs were incubated at 37°C for 18 hours before supernatants were harvested. IMQ was used at 100 μg/mL as a positive control. When HCQ and ODN (Miltenyi Biotec, catalog 130-105-820) were used, both were used at 5 μM. IFN-α production was measured with the Human IFN-alpha 2/IFNΑ1 DuoSet ELISA (R&D Systems, catalog DY9345-05) according to the manufacturer’s protocol.

### HEK-hTLR7 stimulation assay.

HEK-Blue-hTLR7 cells with a SEAP reporter gene (InvivoGen, catalog hkb-htlr7) and the parental HEK-Blue Null1-k cells (InvivoGen, catalog hkb-null1) were cultured according to the manufacturer’s instructions. IMQ (MilliporeSigma, catalog 99011-02-6) at increasing concentrations from 0.2 μg/mL to 1,000 μg/mL was used to verify the ability of hTLR7 cells to respond to TLR7 ligand in the media. A total of 1 × 10^4^ HEK-hTLR7 cells were seeded in 100 μL RPMI (Thermo Fisher Scientific, catalog 11875093) the day before transfection in a 96-well plate. Each well was treated with 100 μg/mL RNA oligonucleotides (XIST1.1, RNA9.2a, RNA9.2s, or polyA) in the presence of 0.1 mM guanosine (MilliporeSigma, catalog G6752-1G) and 10 U SUPERase-In RNase Inhibitor. For transfection experiments, transfection using Lipofectamine MessengerMAX reagent (Thermo Fisher Scientific, catalog LMRNA003) was conducted according to the manufacturer’s instructions. Cells were incubated for 24 hours at 37°C with stimuli, and SEAP production was measured at 616 nm using a spectrophotometer. Fold induction was calculated as Δ_A_/Δ_0_, with Δ_A_ equal to the difference in absorbance between the treated well A and an untreated well, and Δ_0_ equal to the difference in absorbance between the comparator (mock transfection or polyA treatment) wells and the untreated wells (28).

### Fluorescence anisotropy.

To test whether XIST1.1 RNA and control oligos bind to TLR7, we performed fluorescence anisotropy using 5′ FAM-labeled customized 20-mer oligos ordered from IDT. Increasing concentrations (10 nM–300 nM) of recombinant Human TLR7 Fc Chimera Protein (R&D Systems; catalog 9567-TR-050) were incubated with 20 nM FAM-labeled RNA oligo for 10 minutes in 50 μL binding buffer (10 μM HEPES, 150 mM NaCl, 300 μM EDTA, 1 mM DTT, and 1% glycerol). Fluorescence polarization was measured using a CLARIOstar^PLUS^ microplate reader (BMG LABTECH) at the Johns Hopkins Center for Molecular Biophysics.

### EMSA.

Interactions between TLR7 and FAM-labeled RNA oligos were analyzed using EMSA. A total of 100 ng of FAM-labeled oligos and 1 μg recombinant TLR7 Fc chimera protein were incubated in 20 μL binding buffer (10 μM HEPES, 150 mM NaCl, 300 μM EDTA, 1 mM DTT, and 1% glycerol) for 30 minutes as previously described (62). After the incubation was complete, 2 μL of Ficoll-Paque Plus (GE Healthcare) was added to each tube, and the reaction product was loaded into a well of a 5% polyacrylamide gel for 20 minutes at 100 V. The FAM-labeled oligos were visualized using a ProteinSimple Fluorochem System, and TLR7 protein on the same gel was detected by Coomassie blue staining.

### CRISPR/Cas9 XIST depletion.

CRISPR/Cas9–mediated depletion of *XIST* was performed using the *XIST* Human Gene Knockout Kit (CRISPR) (Origene, catalog KN412685) according to the manufacturer’s protocol. WT A431 cells (ATCC, catalog CRL-1555) were used as target cells. After the initial round of treatment and serial dilution to generate the XIST-A cell population, a second round of treatment with a different guide RNA (AGCGCTTTAAGAACTGAAGG) in a complex with TrueCut Cas9 Protein (Thermo Fisher Scientific) using Lipofectamine CRISPRMAX (Thermo Fisher Scientific) and serial dilution was performed to generate the XIST-B population. XIST-KO clones were isolated from the XIST-B population by plating cells in 96-well plates at single-cell density. Viable clones were then analyzed for XIST expression using qPCR and RNAScope (ACD Bio/Bio-Techne), leading to the generation of a XIST-KO cell population.

### qPCR.

Total RNA was isolated from WT A431, Jurkat (ATCC), and XIST-KO cell populations using RNeasy Mini Kit (QIAGEN, catalog 74104) with on-column DNase digestion using the RNase-Free DNase Set (QIAGEN, catalog 79254). cDNA was synthesized using SuperScript VILO Master Mix (Invitrogen, catalog 11755050). XIST (Hs01079824_m1 FAM) and GAPDH (Hs99999905_m1 VIC PL) gene expression was detected using TaqMan gene expression assays (Thermo Fisher Scientific), and gene expression was measured using QuantStudio I 3 Real-Time PCR System (Thermo Fisher Scientific, catalog A28136). Samples were measured in triplicate, and the expression of *XIST* relative to the *GAPDH* housekeeping control was calculated using the ΔΔCt method (QuantStudio Design & Analysis Software; Thermo Fisher Scientific). Relative quantity of XIST RNA released was also calculated as 2^ΔCt^ × R, where R was the ratio of RNA isolated from UV-irradiated cells versus live cells.

### RNAScope.

The RNAScope Multiplex Fluorescent V2 Assay (ACDBio, catalog 323132) was performed according to the manufacturer’s protocol for cultured adherent cells. The *XIST* probe was fluorescent in Channel 2 (catalog 311231-C2). Akoya Biosciences fluorophore Opal 520 (Product Code FP1487001KT) was used in conjunction with the RNAScope kit. Cells were visualized on a Zeiss LSM 880 confocal microscope with Airyscan FAST Module at the Johns Hopkins Institute for Basic Biomedical Sciences Microscope Facility.

### RNA isolation and Mg^2+^ RNA fragmentation.

RNA was isolated from A431 cell lines using TRIzol Reagent (Invitrogen, catalog 15596026) according to the manufacturer’s protocol. Fragmentation of A431 cellular RNA was performed using the NEBNext Magnesium RNA Fragmentation Module (New England Biolabs, catalog E6150S). We incubated 2 μg of total RNA with RNA Fragmentation Buffer at 94°C for 20 minutes. Fragmentation was verified using an Agilent 2100 Bioanalyzer according to the manufacturer’s instructions.

### RNA sequencing.

RNA extraction, sequencing, and analysis were performed by the Johns Hopkins The Single Cell & Transcriptomics Core. Single-ended 100-nucleotide sequencing libraries were prepared using the Illumina Stranded Total RNA Prep Ligation with Ribo-Zero Plus total RNA protocol (catalog 20040525). Purified fragmented total RNAs first underwent quality control (QC) and quantification using the Thermo Fisher Scientific NanoDrop 1000 instrument, then underwent rRNA depletion, cleanup, and reverse transcription into cDNA. Barcoded adaptors were ligated to create libraries that were amplified for sequencing on the Core’s Illumina NovaSeq 6000 system. Resulting FASTQ reads were imported into the Partek Flow platform and aligned to NCBI *Homo sapiens* genome GRCh38.p13 RefSeq v99 transcriptome. Gene expression levels were calculated as FPKM. FPKM values were imported into the Partek Genomics Suite 6.6 for statistical analysis. These values underwent QC and were then log_2_-converted and quantile-normalized for comparison across the 3 biological classes using the 1-way ANOVA test to determine differential expression as fold-change and its statistical significance as *P* values for all transcripts. Annotated transcripts that demonstrated adequate FPKM expression levels (log_2_ > 7.0) were used to assess differential expression. Since genes’ log_2_ fold-changes showed a normal distribution, their SDs from unchanged were determined on the Spotfire Genomics Suite v9.1.2 platform (TIBCO), then used to establish the cutoffs for differential expression (e.g., the log_2_ fold-changes > 2SD either up or down for 3 cell class comparisons). These 2SD cutoffs were employed to perform pathway and functional analyses using QIAGEN Ingenuity Pathway Analysis.

RNA-sequencing data were analyzed using Microsoft Excel and R (55). A Benjamini-Hochberg correction was applied to *P* values using Excel. UUs for all transcripts of interest were counted using R and the RefSeq Human Transcriptome.

### AMP data analysis.

The AMP data set was downloaded from ImmPort using SDY997 (63). The Seurat package was used for analysis (64). The raw expression data were pulled from the gene_by_cell_exp_mat.736297.txt file. The celseq_meta file was used to identify which cells belonged to each patient identification number. Patients who contributed fewer than 10 cells to the published RNA-sequencing data set were excluded from the analysis. Seurat was used to normalize and scale the data and to calculate IFN scores via the AddModuleScore function, and the IFN score was defined as previously described (37, 65). Contaminating epithelial cells were removed from the analysis.

### IFN induction.

HEK293 cells, A431 cells, SLE patient PBMCs, or primary keratinocytes (Lifeline Cell Technology) (1 × 10^6^ cells/mL in a 12-well plate) were treated with 1,000 U/mL IFN-α 2a (PBL Assay Science, catalog 11100) and 50 ng/mL IFN-γ (PBL Assay Science, catalog 11500) for 24 hours at 37°C before RNA isolation.

### EV RNA isolation.

For UV irradiation experiments, 80% confluent A431 cells cultured in a 100 mm Petri dish were irradiated under UVB for 1 minute, 40 seconds, at room temperature and then incubated at 37°C for 4 hours before isolation of EVs. EV RNA was isolated using the QIAGEN exoRNeasy exosome/extracellular vesicle isolation kit (catalog 77144). EV RNA was quantified by NanoDrop 2000 (Thermo Fisher Scientific) and used for cDNA synthesis for qPCR.

### Human patients.

Patients with SLE met the American College of Rheumatology criteria for an SLE diagnosis (66–68). Healthy controls were included if they were 18 years of age or older; were not pregnant; did not have a history of autoimmune disease, cancer, or Lyme disease; and did not have active HIV, tuberculosis, or hepatitis infection. Demographic and clinical information were collected at the same visit as sample collection, including the PGA and SLEDAI disease activity measures (Supplemental Table 4). *XIST* expression levels were not available to the doctor assigning the PGA and SLEDAI scores. Blood was collected by venipuncture and processed for downstream assays within 2 hours of collection. PBMCs were isolated from whole blood by Ficoll density-gradient centrifugation (Ficoll-Paque Plus, GE Healthcare).

### RNA flow cytometry.

RNA PrimeFlow (Thermo Fisher Scientific) was conducted per the manufacturer’s protocol with probe sets for human XIST (catalog 68114) and human RPL13A (catalog 63229). Prior to performing PrimeFlow, cells were stained with cell lineage surface markers including CD3 AF700 (Invitrogen, catalog 56-0038-42), CD14 PE-eF610 (Invitrogen, catalog 61-0149-42), CD19 PE-Cy7 (Invitrogen, catalog 25-0199-42), and LIVE/DEAD Fixable Blue Dead Cell Stain viability dye (Invitrogen, catalog L34962), per the protocol. Samples were analyzed using a FACSAria II Flow Cytometer (BD Biosciences) and FCS Express 7 software (De Novo Software). After gating on singlets and live cells, a gate was created for all cells positive for the housekeeping gene RPL13A. From all RPL13A^+^ single cells, gates were created for CD3^+^, CD14^+^, and CD19^+^, defining all T cells, monocytes, and B cells, respectively. Jurkat and HEK293T cells were run in parallel with patient and healthy control cells for several samples in multiple batches to verify that any signal in male cells was above background.

### Statistics.

GraphPad Prism 8.4.3 was used to generate dot plots, bar graphs, and line plots and to conduct 1-way ANOVA tests, 2-tailed *t* tests, and simple linear regression tests. A Benjamini-Hochberg correction for the *P* values of genes in RNA-sequencing data was done in Microsoft Excel. Two-sided *P* values less than 0.05 were considered significant.

### Study approval.

This study included 11 women with SLE recruited from the Johns Hopkins Lupus Center (IRB study number NA_00039294), as well as 12 female and 10 male healthy controls enrolled in an observational study of healthy individuals at Johns Hopkins (IRB00066509). All samples were obtained under informed written consent, and studies were approved by the Johns Hopkins Institutional Review Board.

### Data availability.

The full RNA-sequencing data set is available under NCBI Gene Expression Omnibus accession GSE201160. Code used to generate Figure 1; Supplemental Table 1; Supplemental Table 3, A and B; and Supplemental Figure 2 is published on GitHub at https://github.com/Jcrawford400/XIST-is-a-sex-specific-reservoir-of-TLR7-ligands (commit ID e4e83a8). The values behind all means reported in the manuscript and points plotted are available in the Supporting Data Values XLS file.

## Author contributions

JDC, HW, BA, and ED conceived the study. JDC, HW, DTZ, RC, CCT, AMC, AAG, DWG, AR, BA, and ED performed formal analysis. JDC, HW, DTZ, and BA performed investigation. JDC, HW, RC, JTS, DTZ, BA, and ED developed methods. JTS, DWG, and MP provided resources. DWG, MP, and ED performed data and sample curation. JDC and MAT performed data visualization. JDC and ED wrote the original draft. JDC, HW, RC, CCT, MAT, AMC, JTS, DTZ, AF, DWG, MP, AR, BA, and ED reviewed and edited the draft. ED supervised. BA and ED acquired funding.

## Supplementary Material

Supplemental data

Supplemental table 1

Supplemental table 2

Supplemental table 3

Supplemental table 3

Supplemental table 5

Supplemental table 5

Supporting data values

## Version 1 09/21/2023

In-Press Preview

## Version 2 10/23/2023

Electronic publication
