# Supplementary material for: The XIST lncRNA is a sex-specific reservoir of TLR7 ligands in SLE
Source: JCI Insight. 2023 Sep 21;8(20):e169344. doi: 10.1172/jci.insight.169344 (PMC10634230; doi:10.1172/jci.insight.169344)
Supplement: Supplemental data [file jciinsight-8-169344-s006.pdf]

Supplementary Data

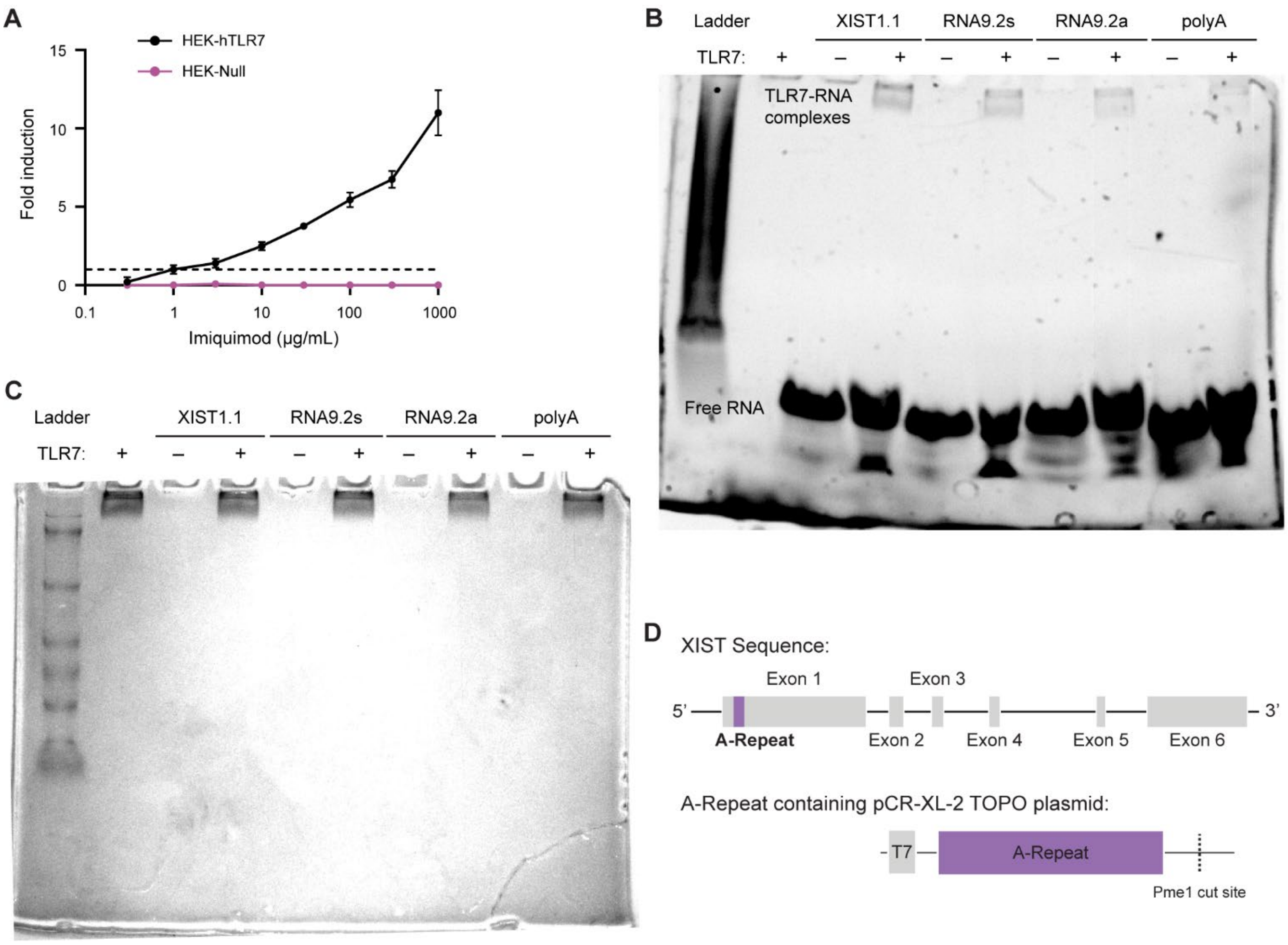

Supplementary Fig. 1 | Cloning the A-repeat region. (A) Colorimetric assay showing the production of SEAP by HEK-hTLR7 and HEK-null cells in response to treatment with varying doses of IMQ in 3 technical replicates. Fold induction relative to absorbance induced by 1 µg/ml IMQ (dotted line). **(B)** Electrophoretic mobility shift assay showing the banding pattern for FAM-labeled XIST1.1, RNA9.2s, RNA9.2a, and polyA RNA. **(C)** Protein only staining of the gel in (B), showing human recombinant TLR7. **(D)** The A-repeat region was cloned into a pCR-XL-2 TOPO plasmid containing a T7 promoter.

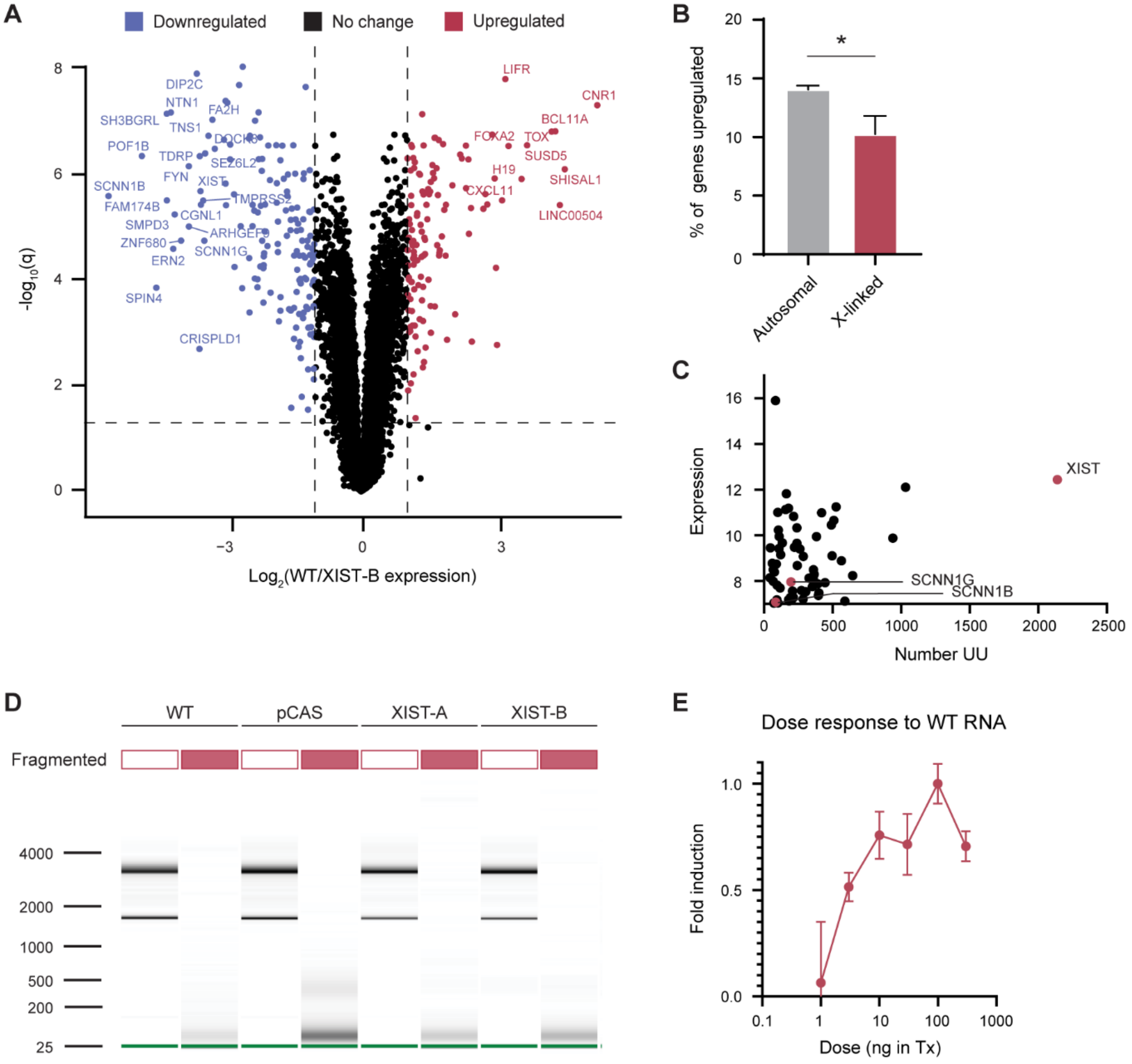

Supplementary Fig. 2 | XIST is the most significantly different RNA enriched in TLR7 ligands between WT and XIST-B cell populations. (A) Volcano plot showing the transcriptional differences between the XIST-B cell population versus WT. **(B)** Bar graph showing the % of genes found to be upregulated in XIST-B cells. Autosomal genes were more often upregulated than X-linked genes. **(C)** Scatter plot showing the difference in expression level and number of UU dinucleotides of all genes “lost” (> 4-fold downregulated with statistical significance) in the XIST-B cell population when compared to the WT. Genes containing the GUCCUUCAA motif are colored (red) and labeled. **(D)** Cellular RNA from A431 cells was fragmented using a Mg^{2+} RNA fragmentation kit prior to transfection. Fragments were 25-50 nucleotides in length. Lanes with fragmented RNA denoted by solid red blocks. **(E)** Colorimetric assay measuring SEAP secretion by HEK-hTLR7 cells transfected with varying concentrations of Mg^{2+}-fragmented cellular RNA from the WT cells.

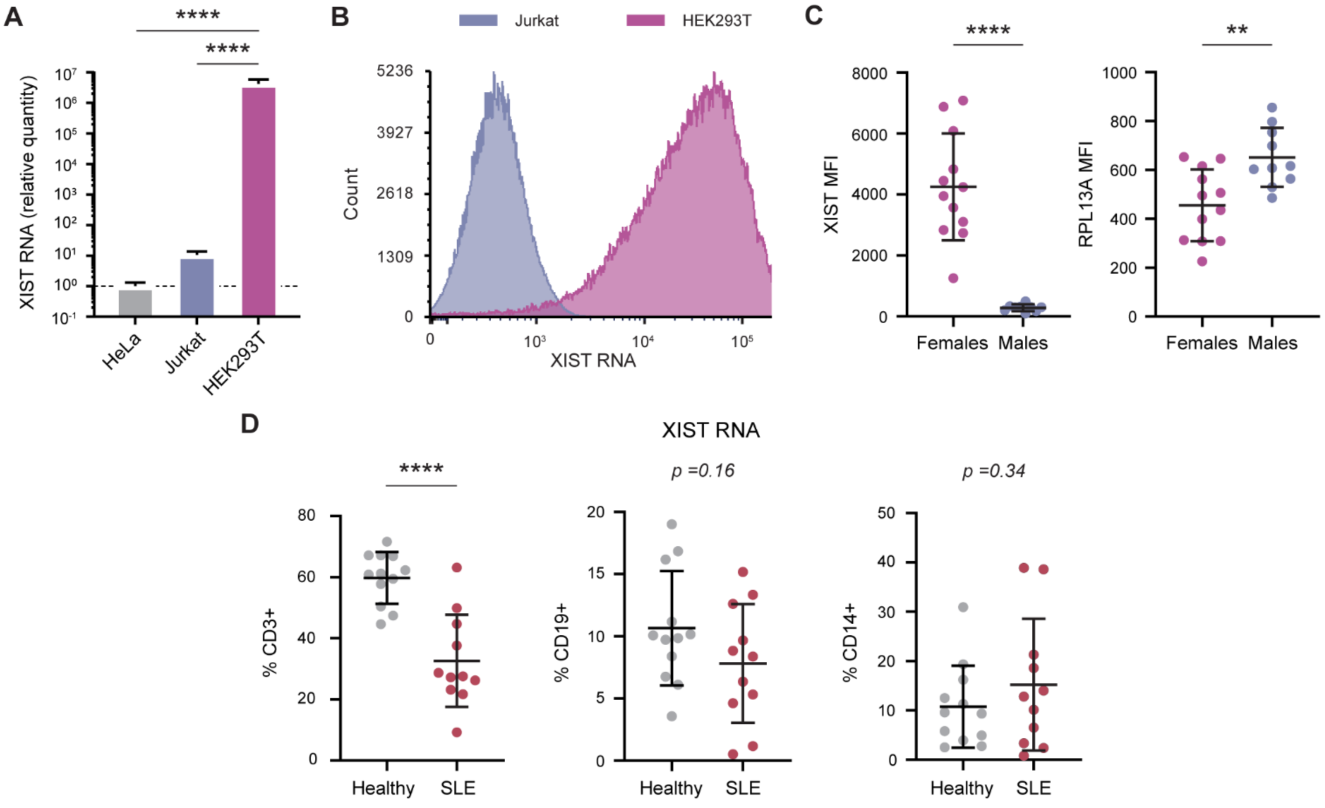

Supplementary Fig. 3 | Validation of RNA PrimeFlow to measure XIST expression.

(A) Bar graphs showing XIST expression as measured by qPCR in HeLa cells (XIST-negative female cell line), Jurkat (male T cell line), and HEK293T cells (a triploid female cell line). HeLa and Jurkat cells compared to HEK293T cells by one-way Anova with multiple comparisons test. **(B)** Histograms of XIST expression in HEK293 cells and Jurkat cells as measured by RNA PrimeFlow. **(C)** Dot plots showing XIST and Rpl13A RNA MFI in female vs. male healthy donors. **(D)** Dot plots showing the frequencies of CD3+ cells, CD19+ cells, and CD14+ cells in Rpl13A+ leukocytes from SLE patients and healthy controls. **(A,C-D)** Error bars represent one standard deviation. **(C-D)** SLE and healthy controls compared by Student's t test. * indicates $p < 0.05$, ** indicates $p < 0.01$, **** indicates $p < 0.0001$.

Supplementary Table 4: Demographics and disease characteristics of SLE patients.

	**SLE (n=11)**	**Healthy Control (n=12)**
Sex		
Female, n (%)	11 (100%)	12 (100%)
Male, n (%)	0 (0%)	0 (0%)
Ethnicity		
Caucasian, n (%)	9 (82%)	10 (83%)
African American, n (%)	2 (18%)	1 (8%)
Native American, n (%)	0 (0%)	1 (8%)
Age at sample collection, years, Mean ± SD	44.5 ± 11.9	37.9 ± 13.2
Age at SLE diagnosis, years, Mean ± SD	25.7 ± 10.9	n/a
Clinical findings		
Malar Rash, n (%)	5 (45%)	n/a
Discoid Rash, n (%)	2 (18%)	n/a
Photosensitivity, n (%)	7 (64%)	n/a
Mouth Ulcer, n (%)	7 (64%)	n/a
Arthritis, n (%)	5 (45%)	n/a
Proteinuria, n (%)	7 (64%)	n/a
Serositis, n (%)	5 (45%)	n/a
Neurologic, n (%)	1 (9%)	n/a
Hematologic, n (%)	10 (91%)	n/a
Immunologic, n (%)	10 (91%)	n/a
ACR Criteria	6.0 ± 1.6	n/a

n/a = not assessed

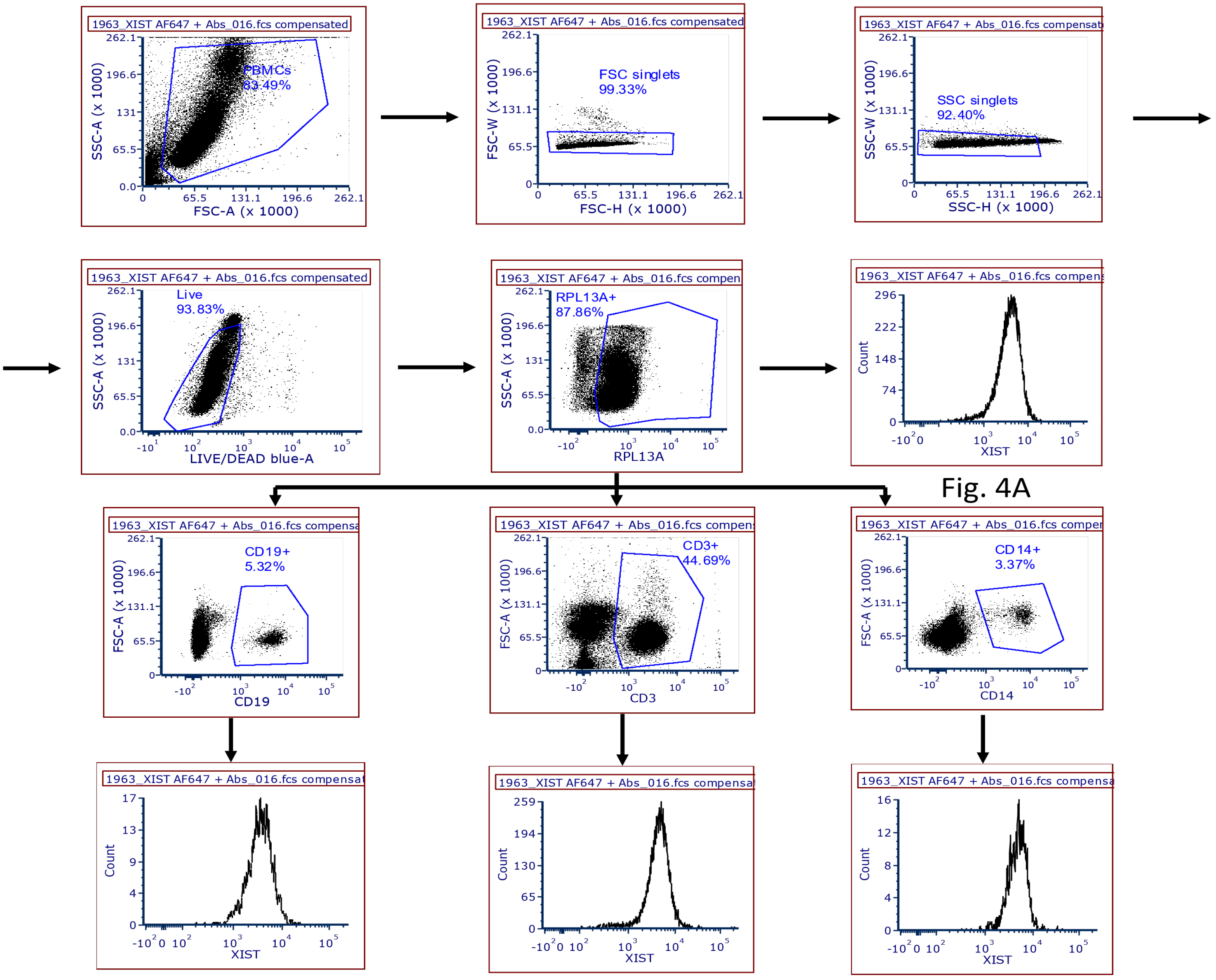

Fig. 4A

Fig. 4B

Fig. 4C

Fig. 4D

Supplementary Fig. 4 | Gating Strategy for RNA Primeflow
